# Thymic Stromal Lymphopoietin Is Implicated in the Pathogenesis of Bullous Pemphigoid by Dendritic Cells

**DOI:** 10.1155/2020/4594630

**Published:** 2020-09-24

**Authors:** Si-Zhe Li, Xin-Xing Jin, Xiao-Lei Ge, Ya-Gang Zuo, Hong-Zhong Jin

**Affiliations:** ^1^Department of Dermatology, National Clinical Research Center for Dermatologic and Immunologic Diseases, Translational medicine Center, Peking Union Medical College Hospital, Chinese Academy of Medical Sciences and Peking Union Medical College, Beijing, China; ^2^Department of Dermatology, Graduate Student of Hebei North University, Zhangjiakou, Hebei, China

## Abstract

**Objectives:**

Both thymic stromal lymphopoietin (TSLP) and dendritic cells (DCs) are involved in many autoimmune diseases, but the potential roles of TSLP and DCs in bullous pemphigoid (BP) have not been clarified. We sought to explore the contributions of TSLP and DCs in patients with BP.

**Methods:**

TSLP levels in sera and blister fluids were measured by enzyme-linked immunosorbent assay. The TSLP expression in the BP lesional skin was detected by immunohistochemical staining. Infiltration of DCs, marked by DC-specific intercellular adhesion molecule-3-grabbing nonintegrin (DC-SIGN), and its relationship with TSLP and TSLP receptors was evaluated by immunofluorescence staining.

**Results:**

We found that TSLP levels in sera and in blister fluids of patients with BP were higher compared to the control groups. In patients with BP, TSLP levels in sera correlated with TSLP levels in blisters. The expression of TSLP in the BP lesional skin was higher compared to the healthy controls' skin. Greater numbers of TSLP-positive cells were observed in the epidermis of patients with BP compared to the healthy controls. Greater numbers of DC-SIGN-positive cells were present in the BP lesional skin compared to the skin of controls. The expression of TSLP was highly upregulated in DC-SIGN-positive cells, and most DC-SIGN-positive cells expressed TSLP receptors.

**Conclusions:**

We conclude that TSLP may activate DC-SIGN-positive DCs directly, which may be involved in the pathogenesis of BP.

## 1. Introduction

Bullous pemphigoid (BP) is the most common autoimmune blistering disease, which typically presents as pruritic vesicles and bullae. Distinguishing histological features of BP include subepidermal blisters accompanied by dermal infiltrates of eosinophils, neutrophils, and mononuclear cells, as well as linear deposition of autoantibodies and complement along the basement membrane zone (BMZ) as measured by immunofluorescence [1]. Autoantibodies to BP230 (BPAg1) and BP180 (BPAg2, collagen XVII), which are two components of the hemidesmosome in the dermal-epidermal junction, can be detected in BP serum [2]. Previous studies suggested that serum levels of pathogenic IgG and IgE autoantibodies against the noncollagenous 16A region (NC16A) of the BP180 ectodomain correlated with the disease activity of BP [2, 3]. Furthermore, multiple kinds of antibodies are produced by B cells, to which helper T (Th) cells provide signals from antigen-presenting cells (APCs).

There are two main types of APCs in the skin: Langerhans cells (LCs), which are predominantly located in the epidermis, and dendritic cells (DCs), which are located in the dermis [4]. LCs were reported to present lipid antigens to Th17 and Th22 cells in skin inflammation [5]. DCs, the most efficient APCs, not only activate T cells but also produce mediators of inflammation that participate in autoimmune diseases [6]. DCs operate a significant part in the pathogenesis of many autoimmune diseases, such as rheumatoid arthritis [7], type 1 diabetes [8], systemic lupus erythematosus [9], and multiple sclerosis [10]. As a typical humoral response and an autoimmune disease, BP should have been influenced by DCs. Previous studies showed that an increased number of DCs was presented in the skin of BP, although a portion of them were defined as LCs [11, 12]. However, it is unknown how DCs or LCs are involved in the pathogenesis of BP. DC-specific intercellular adhesion molecule-3-grabbing nonintegrin (DC-SIGN), also known as CD209, is a kind of innate immune receptor that is expressed on the surface of monocyte-derived DCs, participates in recognizing and capturing antigens, and is involved in many skin diseases, such as atopic dermatitis and psoriasis vulgaris [13–15]. Thus, it would be valuable to investigate the potential role of DC-SIGN-positive DCs in the mechanism of BP.

Thymic stromal lymphopoietin (TSLP), an IL-7-like cytokine, participates in several autoimmune diseases, such as eosinophilic esophagitis, inflammatory bowel disease, and rheumatoid arthritis [16, 17]. In skin inflammation, TSLP is important at the initial step and the late phase of inflammation [18]. Recently, Zhang et al. found production of TSLP by keratinocytes was increased in experimental mice that expressed a form of BP180 that lacked the NC16A domain. This form of BP180 also induced distinctive pruritus in mice, which is one of the distinguishing symptoms of BP [19]. Moreover, increased levels of TSLP have been found not only in experimental mice but also in sera and lesions of patients with BP [19–21]. Furthermore, TSLP acts on DCs as an adjuvant to promote germinal center reactions and Th2 immune reactions, which enhance and shift antibody synthesis [22, 23]. However, the potential roles of TSLP and DCs in BP still need to be clarified.

In this study, we detected TSLP levels in sera and blister fluids and further determined the TSLP expression, DC-SIGN-positive DCs, and LCs in the skin of patients with BP and healthy controls (HCs). Our result suggested that TSLP may potently activate DC-SIGN-positive DCs in the pathogenesis of BP, which will be helpful for our further in vivo investigation to clarify the mechanism.

## 2. Materials and Methods

### 2.1. Patients and Controls

We retrospectively assessed 35 Chinese patients with BP from 2016 to 2018, in the Dermatological Department of Peking Union Medical Collage Hospital (Table 1). These patients were diagnosed according to the following criteria: typical lesions (tense vesicles and bullae on the erythematous skin, with prominent pruritus), dermatopathologic manifestations (subepidermal blister formation with infiltration of neutrophils, eosinophils, and lymphocytes), direct immunofluorescence features (linear deposits of IgG and C3 along the basement membrane zone), and serologic features (anti-BP180 antibody positive, or indirect immunofluorescence indicating deposits of IgG in the epidermal roof of normal human skin, split by 1 M saline solution). HCs were recruited from patients who were diagnosed as having nevus, cysts, or seborrheic keratosis in the same site during the same period (Table 1). This research was reviewed and approved by the Ethical Review Committee of Peking Union Medical College Hospital (ZS-1735). All of the participants were informed of the benefits and risks about enrolling in this research and signed consents for this research. Potential participants who received systematic treatment with glucocorticoids, immunosuppressants, biologics, intravenous immunoglobulins, or plasma exchange were excluded from the study.

Serum samples were obtained from patients with BP (*n* = 35) and HCs (*n* = 44); blister fluids were taken from patients with BP (*n* = 35) and patients with other blister dermatoses (*n* = 39), such as eczema, toxic epidermal necrolysis, contact dermatitis, herpes, and burns (Table 1). The supernatants of blister fluids were prepared for further tests. Serum samples and blister fluids were stored at -80°C before use. Skin tissues were taken from the new-onset blister lesions of patients with BP without topical therapy (*n* = 10) and leftover tissue that remained after excision of nevus or cysts (*n* = 9). The collected tissues were fixed in 10% neutral buffered formalin for 20 h at room temperature, embedded in paraffin, and then sectioned at 6 *μ*m. Prior to immunohistochemistry and immunofluorescence, the tissues were deparaffinized in xylene, rehydrated in a series of graded ethanol, and then retrieved by heat treatment.

### 2.2. Measurement of TSLP Levels in Sera and Blisters by Enzyme-Linked Immunosorbent Assay (ELISA)

TSLP levels in sera and blister fluids were measured using the Human TSLP Quantikine ELISA Kit (R&D Systems, Minneapolis, MN, USA), according to the manufacturer's recommendations. Optical densities were measured at 450 nm and 540 nm using the Varioskan Flash Multimode Reader (Thermo Fisher Scientific, Waltham, MA, USA).

### 2.3. Immunohistochemistry

Paraffin sections were deparaffinized and were then blocked with 3% hydrogen peroxide for 15 min, followed by incubation with the goat serum solution for 1 h at room temperature. Next, rabbit anti-human TSLP (1 : 400, Proteintech Group, Rosemont, IL, USA) was added and incubated overnight at 4°C, followed by incubation with HRP-conjugated affinipure goat anti-rabbit IgG (Proteintech Group, Rosemont, IL, USA). The specimens were then stained with DAB (Solarbio, Beijing, China), followed by washing and counterstaining with hematoxylin (Solarbio, Beijing, China).

For quantitative image analysis, the slides were scanned using NanoZoomer 2.0-RS (Hamamatsu, Japan), and five visual fields were selected randomly for each slide. The number of positive cells was counted at ×40 magnification by four dermatologists separately, and each slide's average number of positive cells was recorded. The staining density of the TSLP expression in the epidermis was converted into a numerical value by using the ImageJ analysis system (Wayne Rasband, National Institutes of Health, USA), a public domain Java image processing program [24].

### 2.4. Double Immunofluorescence

Paraffin sections were deparaffinized and were then blocked with bovine serum albumin (10%) for 1 h at room temperature. Next, primary antibodies, which consisted of mixtures of either mouse anti-human langerin (1 : 50, Santa Cruz Biotechnology, Dallas, TA, USA) or mouse anti-human DC-SIGN (1 : 50, Santa Cruz Biotechnology, Dallas, TA, USA) with rabbit anti-human TSLP (1 : 1,000, Abcam, USA) or goat anti-human TSLP receptor (1 : 13, TSLPR, R&D Systems, Minneapolis, MN, USA), respectively, were added, followed by overnight incubation at 4°C. FITC-labeled donkey anti-mouse IgG (1 : 50, Abcam, USA), AF647-labeled donkey anti-goat IgG (1 : 200, Abcam, USA), and AF647-labeled donkey anti-rabbit IgG (1 : 200, Abcam, USA) were used as secondary antibodies, which were incubated for 1 h at room temperature. Finally, the sections were incubated with 4,6-diamino-2-phenyl indole (DAPI, 0.1 *μ*g/ml, Solarbio, Beijing, China) for 5 min. Slides were viewed in the Vectra 3.0 Automated Quantitative Pathology Imaging System (PerkinElmer, USA).

### 2.5. Statistical Analysis

The number of positive cells in the skin and the density of the TSLP expression in the epidermis were presented as the means ± standard deviations. TSLP levels in sera or blister fluids and the TSLP expression in the skin were expressed as medians and interquartile ranges due to lack of normal distribution, according to the Shapiro-Wilk test. Student's *t* test was used to compare the number of positive cells in the skin and the density of the TSLP expression in the epidermis between groups. The Wilcoxon rank test was used to compare TSLP levels and expression in the skin between groups. The Wilcoxon signed-rank test was used to assess differences in TSLP levels between serum samples and blister fluids of patients with BP. Correlations between variables were evaluated using the Spearman rank correlation test. A *P* value less than 0.05 was considered to be statistically significant. SAS 9.4 (SAS Institute, Cary, NC, USA) was used for statistical analysis. Prism 7 software (GraphPad Software Inc., La Jolla, CA, USA) was used to generate statistical graphs.

## 3. Results

### 3.1. TSLP Levels in Sera and Blister Fluids of Patients with BP Were Higher than in Controls

**Figure 1:**
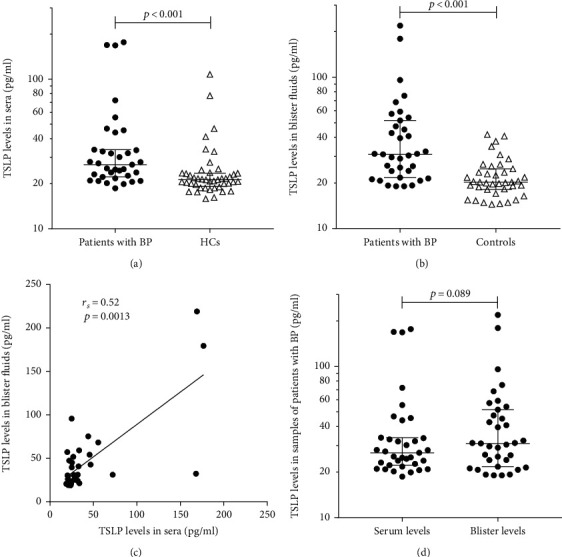
TSLP levels in sera and blister fluids of patients with BP were higher than those in controls. (a) The levels of TSLP in BP sera (*n* = 35) were higher than those in HCs (*n* = 44, *p* < 0.001). (b) The levels of TSLP in blister fluids of patients with BP (*n* = 35) were higher compared to those of other blister dermatoses (*n* = 39, *p* < 0.001). (c) The levels of TSLP in sera correlate with those in blister fluids (*n* = 35, *r*_*s*_ = 0.52, *p* = 0.0013). (d) The levels of TSLP in blister fluids were slightly higher than those in sera, but no significant differences were detected (*p* = 0.089). TSLP levels in sera and blister fluids were investigated by enzyme-linked immunosorbent assay. *p* values were determined by the Wilcoxon rank test in (a, b) or the Wilcoxon signed rank test in (d). Correlation was determined by the Spearman rank correlation test in (c). A *p* value of less than 0.05 was considered statistically significant. Column scatter graphs show the median (lower quartile, upper quartile), and the measured values from individual patients were plotted by dots. TSLP: thymic stromal lymphopoietin; BP: bullous pemphigoid; HCs: healthy controls.

ELISAs were performed in order to examine TSLP levels in sera and blister fluids of patients with BP and controls (Figure 1). The levels of TSLP in BP sera were higher than those in HCs [26.84 vs 22.21 pg/ml, *p* < 0.001Figure 1(a)]. The levels of TSLP in blister fluids of patients with BP were higher compared to those in other blister dermatoses [30.93 vs 20.23 pg/ml, *p* < 0.001; Figure 1(b)]. The levels of TSLP in sera correlated with levels in blister fluids [*r*_s_ = 0.52, *p* = 0.0013; Figure 1(c)]. The levels of TSLP in blister fluids were slightly higher than those in sera, but no significant differences were detected [30.93 vs 26.83 pg/ml, *p* = 0.089; Figure 1(d)]. These results indicated that TSLP is associated with BP, and it may be produced from the skin, although the difference in levels of TSLP between blister fluids and sera was slight and nonsignificant.

### 3.2. The Expression of TSLP in the BP Lesional Skin Was Higher Compared to That in the Control Skin

**Figure 2:**
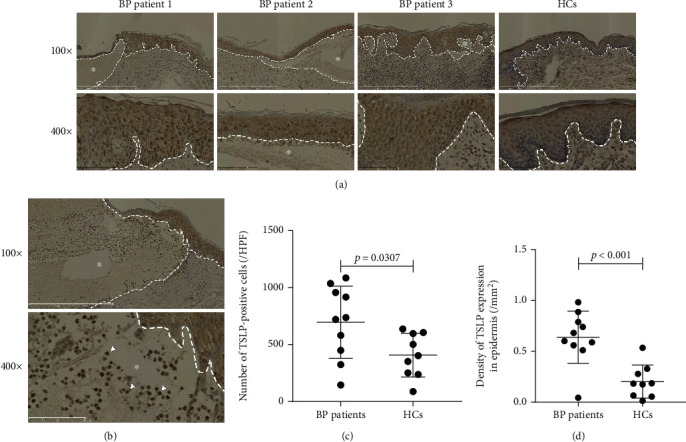
The expression of TSLP in the skin of patients with BP was higher compared to the HC skin. The TSLP expression in the skin of patients with BP and HCs was determined by immunohistochemistry. (a) Compared with the HC skin, the BP lesional skin showed a higher expression of TSLP mainly in the epidermis. (b) TSLP-positive cells (white arrowheads) in blisters of the BP lesional skin. (c) Number of TSLP-positive cells is greater in the BP lesional skin (*n* = 10) compared to the HC skin (*n* = 9, *p* = 0.0307). The number of each section is the average of counts calculated by four dermatologists in five randomly visual fields at HPF (0.068 mm^2^, ×400 magnification) of scanned sections. (d) Density of the TSLP expression in the epidermis of patients with BP was significantly higher than that in the HC epidermis (0.6379 vs 0.2019/mm^2^, *p* < 0.001). Density of the TSLP expression in the epidermis was scored by the ImageJ analysis system as described in the text. Results from three patients with BP and a HCs are shown in (a). Dashed white lines in (a, b) indicate the basement membrane zone or edge of blister. Asterisks (^∗^) indicate the area of blisters. Original magnification: ×100 and ×400. *p* values were determined by Student's *t* test in (c, d). A *p* value of less than 0.05 was considered statistically significant. Column scatter graphs show the mean ± standard deviation, and the measured values from individual patients were plotted by dots. TSLP: thymic stromal lymphopoietin; BP: bullous pemphigoid; HCs: healthy controls, HPF: high power field.

To evaluate the expression of TSLP in the skin, we measured it in patients with the BP lesional skin and HCs' skin using immunohistochemical technology (Figure 2). Compared with the HCs' skin, the expression of TSLP was found to be obviously increased in the BP lesional skin (Figure 2(a)). One notable finding was that the TSLP was mainly expressed in the spinous layer and basal layer of the epidermis in the BP lesional skin, even including cells in blisters (Figures 2(a) and 2(b)), which probably suggested keratinocytes is the main resource of TSLP in BP. In contrast, in the HCs' skin, TSLP was mildly expressed in the basal layer (Figure 2(a)). In addition, there were a few positive cells in the dermis of both the BP lesional skin and HCs' skin. To qualify the expression of TSLP in the epidermis, we counted the number of TSLP-positive cells via four dermatologists separately and measured the density of the TSLP expression in the epidermis using an ImageJ system, as described above. Compared with that in the HCs' skin, there was a statistically significant increase in the TSLP expression in the BP lesional skin [695.18 vs 408.08/HPF, *p* = 0.0307, Figure 2(c)]. Compared with the epidermis of the HCs' skin, the density of the TSLP expression was found to be markedly increased in the epidermis of the BP lesional skin [0.6379 vs 0.2019/mm^2^, *p* < 0.001, Figure 2(d)]. These findings further indicated that TSLP in the skin may be produced by keratinocytes of the epidermis.

### 3.3. DC-SIGN-Positive DCs Infiltrated the Dermis of the BP Lesional Skin More Extensively Than That of the HC Skin

**Figure 3:**
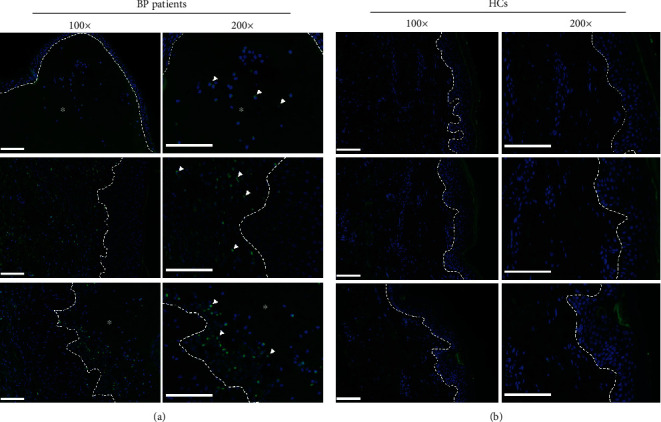
DC-SIGN-positive DCs infiltrated in the dermis of the skin from patients with BP more extensively than those of the HCs' skin. (a) Staining with DC-SIGN (green) and DAPI (blue) showed DC-SIGN-positive DCs (white arrowheads) distinctively infiltrated in the dermis of the BP lesional skin. (b) In the skin of HCs, there are hardly any DC-SIGN-positive DCs. Results from three patients with BP and three HCs are shown in (a, b), respectively. Dashed white lines indicate the basement membrane zone or edge of blister. Asterisks (^∗^) indicate the area of blisters. Original magnification: ×100 and ×200, white scale bar = 100 *μ*m. BP: bullous pemphigoid; DAPI: 4,6-diamino-2-phenyl indole; DCs: dendritic cells; DC-SIGN: dendritic cell–specific intercellular adhesion molecule-3-grabbing nonintegrin; HCs: healthy controls.

**Figure 4:**
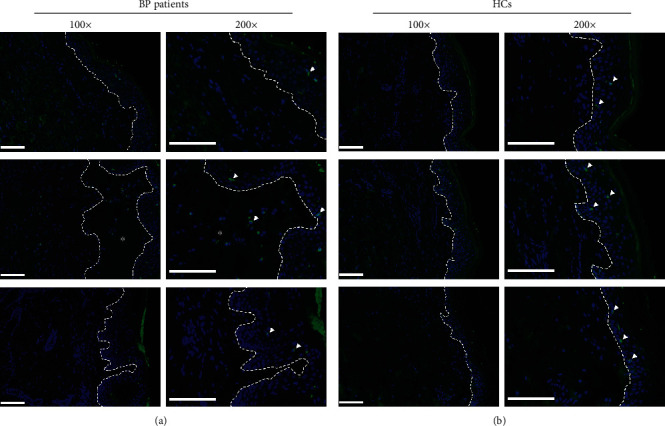
Langerin-positive LCs infiltrated in the skin of patients with BP and HCs without difference. (a) Staining with langerin (green) and DAPI (blue); langerin-positive LCs (white arrowheads) infiltrated in the epidermis of the lesional skin in patients with BP. (b) Langerin-positive LCs (white arrowhead) also infiltrated in the epidermis of the HCs' skin. Results from three patients with BP and three HCs are shown in (a, b), respectively. Dashed white lines indicate the basement membrane zone or edge of blister. Asterisks (^∗^) indicate the area of blisters. Original magnification: ×100 and ×200, white scale bar = 100 *μ*m. BP: bullous pemphigoid; DAPI: 4,6-diamino-2-phenyl indole; HCs: healthy controls; LCs: Langerhans cells.

To analyze APCs in BP, we next stained DC-SIGN or langerin in five BP lesional skins and five HCs' skins by immunofluorescence (Figures 3 and 4). The DC-SIGN-positive DCs infiltrated extensively in BP lesional skins, mainly in the papillary dermis and surrounding the vessels (Figure 3(a)), but no DC-SIGN-positive DCs were found in the HCs' skin (Figure 3(b)). However, in both the BP lesional skin (Figure 4(a)) and HCs' skin (Figure 4(b)), many langerin-positive LCs were found within the epidermis, and there were no obvious differences in the amount of LC infiltration in both kinds of samples. These results supported the conclusion that DC-SIGN-positive DCs, but not LCs, may be involved in the mechanism of BP.

### 3.4. The Expression of TSLP in the BP Lesional Skin Was Associated with the Amount of DC-SIGN-Positive DCs

**Figure 5:**
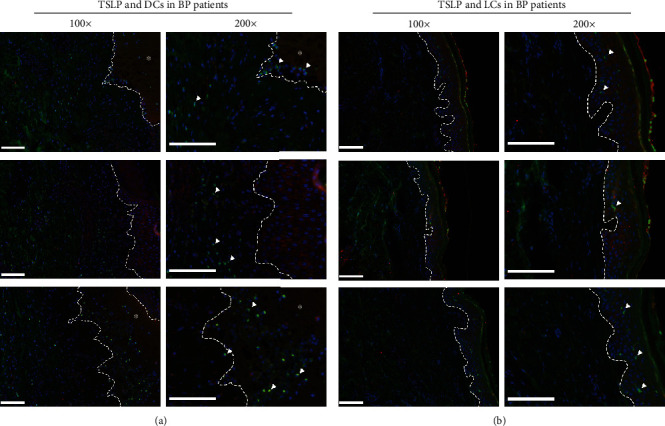
The expression of TSLP in the BP lesional skin was associated with the amount of DC-SIGN-positive DCs, but not LCs. (a) Staining with DC-SIGN (green), TSLP (red), and DAPI (blue) showed the amount of DC-SIGN-positive DCs (white arrowheads) infiltrated in the dermis beneath blisters or TSLP-positive epidermis. (b) Staining with langerin (green), TSLP (red), and DAPI (blue) showed that the LCs (white arrowhead) existed in the epidermis regardless of whether TSLP was positive or not. Results from three patients with BP are shown in (a, b), respectively. Dashed white lines indicate the basement membrane zone or edge of blister. Asterisks (^∗^) indicate the area of blisters. Original magnification: ×100 and ×200, white scale bar = 100 *μ*m. BP: bullous pemphigoid; DAPI: 4,6-diamino-2-phenyl indole; DCs: dendritic cells; DC-SIGN: dendritic cell–specific intercellular adhesion molecule-3-grabbing nonintegrin; LCs: Langerhans cells; TSLP: thymic stromal lymphopoietin.

To investigate whether the TSLP expression in BP is associated with APCs, TSLP was stained with either DC-SIGN or langerin by immunofluorescence experiment on slides of five patients with BP and five HCs (Figure 5). Stronger TSLP expression in the epidermis or cells in blisters of the BP lesional skin was associated with the concurrent appearance of DC-SIGN-positive DCs (Figure 5(a)). In contrast, LCs existed in the epidermis of the lesional skin, regardless of whether or not the TSLP expression was strong (Figure 5(b)). These results implied that TSLP produced by keratinocytes in the BP lesional skin may cooperate with DC-SIGN-positive DCs on the mechanism of BP.

### 3.5. TSLP Affects the DC-SIGN-Positive DC Infiltration Directly in the BP Lesional Skin

**Figure 6:**
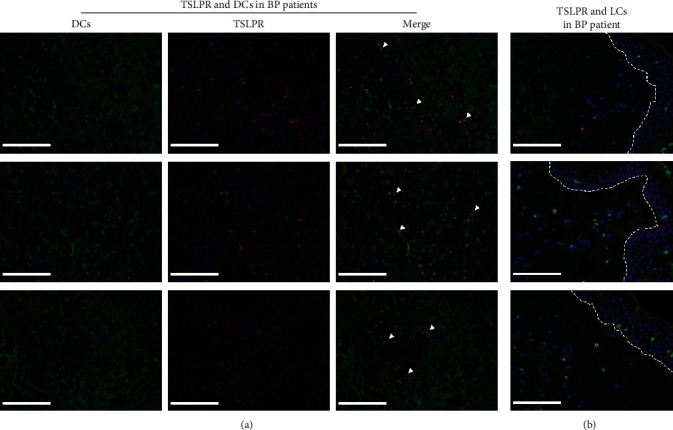
DC-SIGN-positive DCs were positive for TSLPR in the BP lesional skin, whereas langerin-positive LCs were not positive for TSLPR. (a) Staining with DC-SIGN (green), TSLPR (red), and DAPI (blue) showed the amount of DC-SIGN and TSLPR double-positive DCs (orange, white arrowhead) infiltrated in the dermis of the BP lesional skin. (b) Staining with langerin (green), TSLPR (red), and DAPI (blue) showed that all of langerin-positive LCs (green, white arrowhead) were negative for the TSLPR (red) expression. Results from three patients with BP are shown in (a, b), respectively. Dashed white lines indicate the basement membrane zone or edge of blister. Asterisks (^∗^) indicate the area of blisters. Original magnification: ×200, white scale bar = 100 *μ*m. BP: bullous pemphigoid; DAPI: 4,6-diamino-2-phenyl indole; DCs: dendritic cells; DC-SIGN: dendritic cell–specific intercellular adhesion molecule-3-grabbing nonintegrin; LCs: Langerhans cells; TSLPR: thymic stromal lymphopoietin receptor.

Finally, to reveal whether TSLP produced by keratinocytes in BP affects DCs' infiltration in the lesional skin directly, TSLPR was stained with either DC-SIGN or langerin by immunofluorescence experiment on slides of five patients with BP (Figure 6). The DC-SIGN-positive DCs were also positive for the TSLPR expression (Figure 6(a)), whereas langerin-positive LCs did not express TSLPR (Figure 6(b)). These observations further support that DCs, but not LCs, may be related to TSLP in the pathogenesis of BP and indicate that TSLP produced by keratinocytes in BP affects DC-SIGN-positive DCs directly and may be involved in the pathogenesis of BP.

## 4. Discussion

Our present study showed that the TSLP expression and DC-SIGN-positive DCs' infiltration increased in the samples of patients with BP, and TSLP might affect DC-SIGN-positive DCs in BP. We identified increasing expression of TSLP in sera, blister fluids, and lesional skin of patients with BP. Our results showed that DC-SIGN-positive DCs infiltrated in the BP lesional skin more extensively, compared to the skin of HCs. Furthermore, in this study, we provided evidence that TSLP was relevant to DC-SIGN-positive DCs in BP, and TSLPR was expressed in DC-SIGN-positive DCs. Together, these data strengthen the hypothesis that the interaction of DC-SIGN-positive DCs-TSLP-TSLPR may be involved in the pathogenesis of BP.

TSLP, as a Th2-related cytokine, was found to be involved in BP recently [19–21]. By investigating TSLP in the BP mouse model and patients, Zhang et al. found high levels of TSLP in sera, blister fluids, and BP lesions of patients with BP [19], and severe pruritus of BP was related to TSLP, whereas in another study, TSLP levels did not correlate with the severity of pruritus in BP, although the expression of TSLP was enhanced in BP lesions compared to HCs [21]. Regardless of whether or not TSLP is associated with itch, it is possible that TSLP is related to BP and could not be ignored in the pathophysiology. Likewise, in our research, the sera of patients with BP contained significantly increased the levels of TSLP, compared to HCs. Similarly, high levels of TSLP were observed in blister fluids of patients with BP, compared to other diseases, such as eczema, toxic epidermal necrolysis, contact dermatitis, herpes, and burn. These studies and our findings indicate that TSLP is involved in BP.

TSLP is produced by various types of cells, such as epithelial cells, keratinocytes, fibroblasts, and DCs [17, 25]. It was previously demonstrated that TSLP is highly produced by skin keratinocytes in dermatitis and psoriasis [26–28]. In the BP lesional skin, TSLP was discovered to be expressed mainly in the epidermis [20, 21]. A study by Zhang et al. showed that dysfunction of BP180 led to the increased expression of TSLP in skin keratinocytes [19]. Although, in our study, there were no significant differences between levels of TSLP in blister fluids and sera, the former was slightly higher than the latter to some extent. This might have been due to the fact that the differences between TSLP levels in sera and in blister fluids were not distinctive or that other unknown factors influenced the expression of TSLP. Our study indicated coherence between TSLP levels in sera and in blister fluids. The immunohistochemistry results further implied that keratinocytes of the lesional skin expressed higher levels of TSLP in patients with BP than in HCs, similar to other diseases [26–28].

Both LCs and DCs, as the main professional APCs of the skin, promote production of antibodies by regulating T follicular helper cells [29, 30]. DCs/LCs involved in BP pathogenesis have been described. Compared to healthy adults, the BP lesional skin contains more LCs/DCs, which may process BMZ antigen [12]. Another study revealed that Fc*ε*RI^+^CD207^−^ cells, which were presumed to be inflammatory dendritic epidermal cells, in the BP lesional skin were significantly higher than those in the perilesional skin of patients with BP and the skin of HCs [31]. In our study, we found that the number of DC-SIGN-positive DCs in the BP lesional skin was greater than that in HCs. Due to the important role of DC-SIGN in antigen presenting, it was reported to bind with many pathogens, such as human immunodeficiency virus, hepatitis C virus, Helicobacter pylori, and Candida albicans [32]. In our present study, we found that there were more DC-SIGN-positive DCs in the lesional skin of patients with BP, which suggested that DC-SIGN-positive DCs were involved in the pathogenesis of BP.

In previous research, the Th2 immune response was shown to likely play a key role in BP [33, 34]. More interestingly, TSLP was presumed to process diseases by orchestrating DCs and Th2-type immune responses [22, 28]. In an asthma study, the DCs that were treated with TSLPR-mAb produced less chemoattractant cytokine ligand 17, which is a type of chemokine that can promote Th2 immune reactions [35]. Our study showed that DC-SIGN-positive DCs in the dermis were in close association with TSLP and that DC-SIGN-positive DCs also expressed TSLPR. These findings imply that interaction between TSLP-TSLPR and DC-SIGN-positive DCs may influence the pathogenesis of BP.

Nevertheless, LCs in the epidermis did not present a clear association with TSLP. Unlike our study, Soumelis et al. [26] found that dermal DCs expressed langerin in atopic dermatitis, which implied that TSLP contributed directly to activating epidermal LCs and promoted LCs migrating to the dermis. Furthermore, it was proved that TSLP directly promotes LCs to release Th2 cytokines and attract chemokines in atopic dermatitis [36]. However, a recent report indicated that TSLP plays an important role in antigen-specific Th cells' proliferation and germinal center B cells' expansion though CD11c-positive DCs, but not LCs, which is similar to our study [37]. In our experiments, LCs also did not express TSLPR, in contrast with some previous studies [36, 37]. On the one hand, epidermal LCs not only originate from a primitive yolk sac but also develop from monocytes and myeloid precursors cells under severe inflammation [38]. The multiple sources of LCs may explain the difference between previous reports and ours in the TSLPR expression of LCs. On the other hand, Nguyen et al. [39] found that the TSLPR expression by LCs used a quantitative polymerase chain reaction, but TSLPR was too weak to be detected either in freshly isolated LCs from the human skin or in immature LC-like cells from the umbilical cord blood through fluorescent dyes, which may account for the different results. Collectively, the precise relationship among TSLP, TSLPR, and LCs, as well as their roles in the pathogenesis of BP, also needs further exploration.

## 5. Conclusions

In summary, our research demonstrated that the levels of TSLP were higher in the lesional skin, blister fluids, and sera of patients with BP than in HCs or other blister dermatoses. DC-SIGN-positive DCs, which infiltrated near the TSLP-positive epidermis and expressed TSLPR, infiltrated the dermis in BP lesion more extensively than the skin of HCs. Together, our findings add to the accumulating evidence that TSLP expressed by epidermal keratinocyte activates DC-SIGN-positive DCs directly, which may be involved in the pathogenesis of BP. A further study is warranted to reveal specific subtypes of DCs activated by TSLP, clarify the relationship between LCs and TSLP, and uncover the downstream mechanisms of TSLP and DCs.

## Figures and Tables

**Table 1:** List of patients with BP, HCs, and controls in experiment of enzyme-linked immunosorbent assay.

Group	Sample type	Number of cases	Age, years [mean (range)]	Sex	
				Male	Female
BP patients	Sera & blister fluids	35	74.14 (55-93)	20	15
HCs	Sera	44	60.09 (50-79)	29	15
Controls	Blister fluids	39	49.46 (19-82)	18	21

Age was recorded at the time of sample collection. BP: bullous pemphigoid; HCs: healthy controls.

## Data Availability

The dataset included in this paper is available from the corresponding author on reasonable request and with appropriate additional ethical approvals, when necessary.
